# Comparision of Ligasure hemorrhoidectomy and conservative treatment for thrombosed external hemorrhoids (TEH) in pregnancy

**DOI:** 10.1186/s12893-023-01910-1

**Published:** 2023-01-19

**Authors:** Huihua Luo, Xiaojun He, Min Wang, Xiaosong Zheng, Rong Peng, Chenwei Wang, Qiu Li, Bolin Yang

**Affiliations:** 1 Department of Colorectal Surgery, Affiliated Hospital of Nanjing University of Chinese Medicine, Nanjing, China; 2 Department of General Surgery, Maternal and Child Health Hospital of Hubei Province, Tongji Medical College, Huazhong University of Science and Technology, Wuhan, Hubei China

**Keywords:** Thrombosed external hemorrhoids (TEH), Ligasure hemorrhoidectomy, Conservative treatment, Recurrence, Satisfaction

## Abstract

**Background:**

Ligasure hemorrhoidectomy for thrombosed external hemorrhoids in pregnancy has been rarely studied.

**Objective:**

The purpose of this article is to study the efficacy and safety of Ligasure hemorrhoidectomy comparing with conservative treatment for thrombosed external hemorrhoids in pregnancy.

**Design:**

This was a retrospective cohort study.

**Setting:**

The patients were treated at a tertiary referral center in China.

**Patients:**

94 pregnant patients hospitalized for thrombosed external hemorrhoids from September 2020 to December 2021.

**Interventions:**

Ligasure hemorrhoidectomy treatment or conservative treatment according to the patient’s wishes.

**Main outcome measures:**

Symptom relief, recurrence and satisfaction of thrombosed external hemorrhoids in pregnancy with different interventions.

**Results:**

There were no differences between groups in maternal age, gestational age, body mass index, parity, constipation and a prior history of thrombosed external hemorrhoids. The pain scores were less in surgical group than in conservative group in post-treatment days 1 and 7. Time to return to normal activities was shorter in surgical group than in conservative group (6.51 vs. 13.52 days, P < 0.001). Post-treatment complications were mild in surgical group and there were no significant differences concerning the rate of abortion, preterm birth, cesarean delivery and weight of fetus. Recurrence rate was significantly lower in surgical group (8.57% vs. 30.43%, P = 0.017). The patient satisfaction scores were significantly higher in surgical group than in conservative group (Z = − 2.979, P = 0.003).

**Limitations:**

This was a retrospective study with a limited number of patients, the data was obtained from only one center.

**Conclusions:**

Comparing with conservative treatment, Ligasure hemorrhoidectomy for TEH in pregnancy results in more rapid pain relief, shorter time to return to normal activities, lower incidence of recurrence, and better patient satisfaction. This type of surgery has low and mild postoperative complications, is not attended by any risk to the mother or her fetus.

## Background

Thrombosed external hemorrhoid (TEH) in pregnancy is the manifestation of the acute attack of hemorrhoid due to the increased intra-abdominal pressure from uterine growth, constipation and diarrhea. TEH, can involve both the external and internal hemorrhoidal plexuses, being the first more frequently affected, a certain degree of hypertonicity of the internal anal sphincter may trap the hemorrhoidal mass outside the anus leading to strangulation, necrosis, and gangrene [1–3]. From acutely intense pain, a visible and can’t returning lump, sometimes combine bleeding when thrombosis leads to the necrosis of overlying skin, we can make the correct diagnosis easily. In fact, these symptoms cause real difficulty in dealing with the activities of everyday life [4].

Hemorrhoidectomy is only indicated in strangulated or extensively thrombosed hemorrhoid in pregnancy [5]. The surgery has been successfully performed without risk to the fetus [6]. In fact, hemorrhoidectomy was sometimes considered risky as it induced bleeding, pain, and serious post-operative complications. Therefore, conservative treatment was the first choice. Nowadays there is deficiency the strong evidence of the efficacy and safety of topical medicine or oral phlebotonics. Conservative treatment usually contains increasing fiber and oral fluid intake, avoiding constipation or diarrhea, assisting by hand for the swelling return, improving toilet habits and so on. During this treatment period, pregnant patients with TEH need to bear the risk of slowly resolution of symptoms and recurrence.

Surgical intervention can be made more effectively and reliably because of development in surgical technique. The usage of the Ligasure technique results in a significantly shorter operative time and less postoperative pain without any adverse effect on postoperative complications, compared to conventional hemorrhoidectomy [7]. Moreover, patients treated with Ligasure hemorrhoidectomy returned to work earlier [8].

In fact, sufficient data existing on the efficacy and safety of Ligasure hemorrhoidectomy for TEH is acquired according to the application of this technology in nonpregnant patients. Ligasure hemorrhoidectomy for TEH in pregnancy has been rarely studied. In this article, we aim to study the efficacy and safety of Ligasure hemorrhoidectomy for TEH in pregnancy.

## Patients and methods

94 pregnant patients with TEH between their 6th and 35th week of pregnancy treated from September 2020 to December 2021 were included. Patients were offered Ligasure hemorrhoidectomy or conservative treatment, and they were allowed to choose between treatment options by themselves. The inclusion criteria were as follows: (1) consistent with clinical manifestation of thrombosed external hemorrhoids (TEH): involve both the external and internal haemorrhoidal plexuses; the haemorrhoidal mass outside the anus leading to strangulation, necrosis, and gangrene; (2) Clinical symptoms: acutely intense pain, painful anal swelling, bleeding, and a visible perianal/anal lump; (3) less than 35 weeks of gestation. The exclusion criteria were as follows: (1) more than 35 weeks of gestation; (2) patients with anal fissure, perianal abscess, fistula, inflammatory bowel disease; (3) immunocompromised patients; (4) cardiac disease, diabetes, cirrhotic patients or after pelvic radiotherapy; (5) patients under anticoagulant therapies; (6) patients with colorectal or anal neoplasia; (7) previous anal surgical procedures.

All patients who underwent therapy performed preoperative laboratory, electrocardiographic testing and fetal heart monitoring. Patients’ characteristics included demographics and clinical characteristics (maternal age, gestational age, BMI, parity, constipation and a prior history of TEH) were obtained. Pre- and post-treatment pain scores, time to return to normal activities, post-treatment complications, pregnancy outcomes, recurrence, and patient satisfaction were all assessed. Patients were followed up in 1 day, 1 week, and 2 weeks post-treatment and had a final follow-up visit in 3 months postpartum.

Clinical evaluation was performed by the surgeons. The Rome IV criteria were used to define constipation and the Bristol Stool Chart (BSC) was used to determine stool type. Patients were asked to complete pain surveys using a visual analogue scale (VAS) ranging from 0 cm (no pain) to 10 cm (the worst pain ever experienced) during the peri-treatment period in days 0, 1, 7, and 14. Patients were advised to return to normal activities as soon as they felt able. Abortion was defined as fetal loss before 28 weeks of gestation. Preterm birth was defined as delivery in the range of 28 to 36 weeks of gestation. Recurrence was defined as the onset of symptoms (pain and bleeding) or new prolapse at follow-up to 3 months postpartum. Patient satisfaction was assessed with 4 categories: excellent (score = 4), good (score = 3), fair (score = 2), and poor (score = 1), in 1 week, 2 weeks post-treatment and had a final follow-up visit in 3 months postpartum. The local Institutional Board of Ethics approved the study (2022IEC057).

### Surgical procedure and technique

Patients in surgical group received spinal anesthesia with 0.5% lidocaine in right lateral decubitus. They were placed in the lithotomy slight left lateral position to prevent uterine compression of the vena cava and left iliac vein and supplemented by monitored anesthesia care and fetal heart care. Ligasure hemorrhoidectomy consisted of excision of both external and internal components using the Ligasure handset up to the anorectal junction without ligation of the pedicle, retaining enough anal margin skin at the same time to avoid postoperative stenosis. When they were freed from anal pain and meanwhile evacuation had occurred, the patients were discharged from the hospital. Patients received regular laxatives for 1 weeks (Polyethylene glycol 4000 10 mg po qd or Lactulose 10 ml po qd) after discharge. Fetal heart monitoring was crucial and done throughout the whole therapy period.

### Conservative treatment

Patients in conservative group received fiber and oral fluid intake, stool softeners (Polyethylene glycol 4000 10 mg po qd or Lactulose 10 ml po qd), assisting by hand for the swelling return, warm sits bath, topical analgesia treatment (0.5% lidocaine cream) when the patients felt severe anal pain, consultation and frequent communication with obstetrical colleagues to help pregnancy refrain from straining and anxiety.

### Statistical analysis

The statistical analysis was conducted using IBM Statistical Package for the Social Sciences (IBM SPSS), version 23.0, software. Analysis of the distribution of the data using the Shapiro–Wilk test, and Levene’s test was applied to confirm the homogeneity of variability between groups. Descriptive quantitative data were displayed in the form of mean, median, standard deviation, range, and interquartile range (IQR), while numerical and percentage values were chosen to describe qualitative data. An independent t-test was performed to compare quantitative data and parameter distributions between two groups. The non-parametric distribution was performed by using the Mann–Whitney test. For the qualitative data, Chi-square tests were carried out to compare between the groups. *P* < 0.05 was considered significant.

## Results

**Fig. 1:**
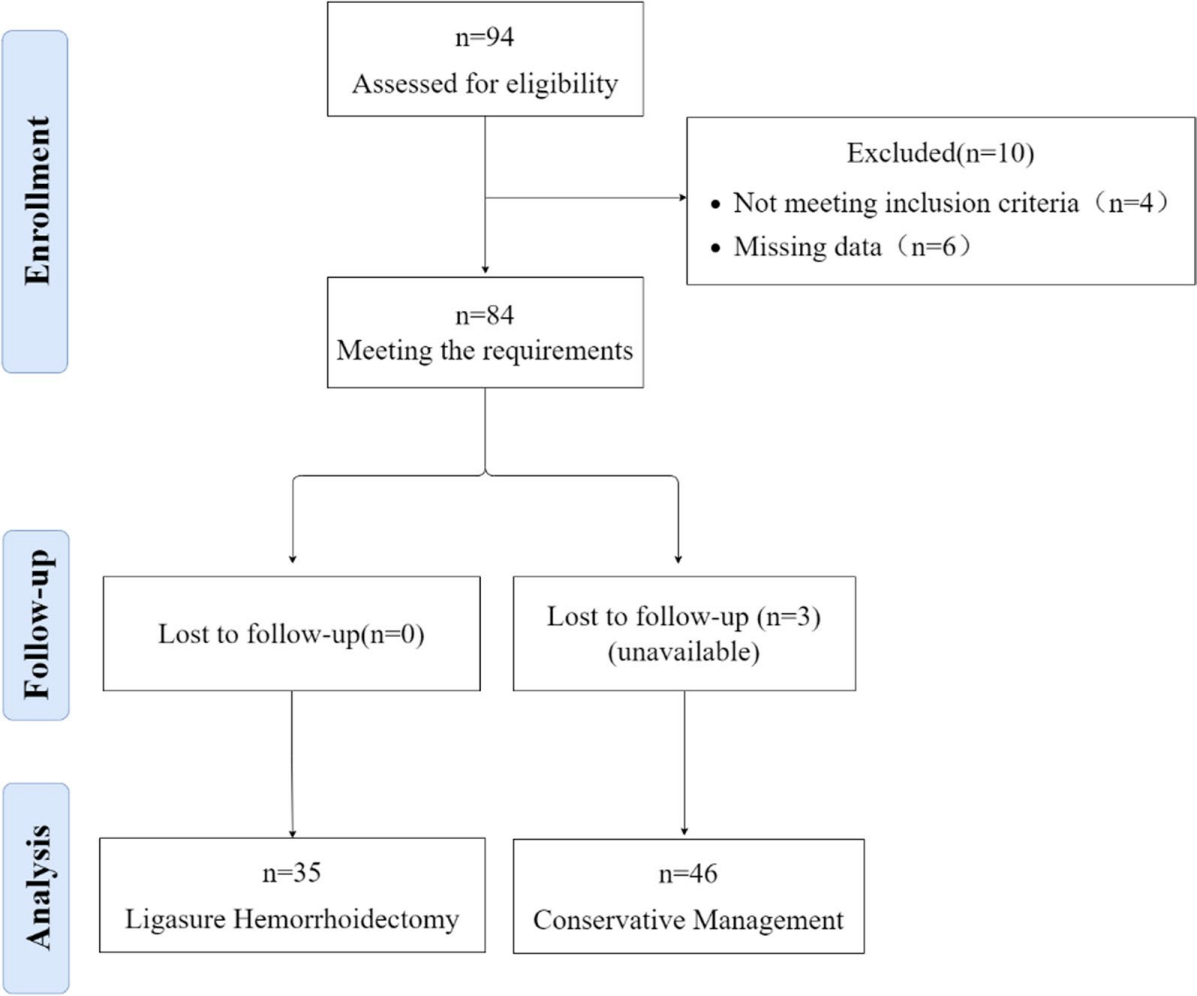
Flow diagram of patients at each stage of treatment THE in pregnancy

94 pregnant patients with TEH between their 6th and 35th week of pregnancy treated from September 2020 to December 2021 were included. 13 patients were excluded for several reasons (Fig. 1), leaving 81 patients for analysis.

Demographics and characteristics were summarized in Table 1. There were no differences between groups in maternal age, gestational age, BMI, parity, constipation and a prior history of TEH. The incidence of most patients (65/81, 80.23%)is concentrated in the last trimester of pregnancy.*46.91%* (38/81) of all patients had constipation, and *38.27%* (31/81) of all patients had a prior history of TEH.

**Table 1:** *Demographics and characteristics* of Ligasure Hemorrhoidectomy and Conservative Treatment for TEH in pregnancy

Characteristic	Ligasure hemorrhoidectomy (n = 35)	Conservative management (n = 46)	*P* value
Age (years)	30.20 ± 3.44^a^	29.00 ± 2.98^a^	0.097
Gestational age (weeks)	28.3(22.5–31.4)^b^	25.75 (19.38–29.53)^b^	0.119
BMI (kg/m^2^)	21.67 (19.5–23.88)^b^	20.53 (19.17–22.93)^b^	0.375
Parity	0 (0–1)^b^	0 (0–1)^b^	0.517
Constipation	18 (51.43%)^c^	20 (43.48%)^c^	0.478
History of TEH	17 (48.57%)^c^	14 (30.43%)^c^	0.096

*BMI* body mass index, *THE* thrombosed external hemorrhoids

^a^Means ± SD

^b^Median (IQR)

^c^n (%)

### Pain scores

There was no difference in pre-treatment pain score of two groups. The median pain scores were less in surgical group than in conservative group in post-treatment days 1 and 7. In post-treatment days 14, there was no obvious difference in two groups (Table 2).

**Table 2:** *VAS scores* of Ligasure hemorrhoidectomy and conservative treatment for TEH in pregnancy

VAS score	Ligasure Hemorrhoidectomy (n = 35)	Conservative Management(n = 46)	*P* value
Pre-treatment (day 0)	8 (6–9)	8 (6–9)	0.954
Post-treatment (day 1)	3 (2–5)	6 (5–7)	< 0.001*
Post-treatment (day 7)	1 (0–2)	3 (2–4)	< 0.001*
Post-treatment (day 14)	0 (0–1)	0 (0–1)	0.657

Results expressed as median (IQR); *Statistically significant

### Time to return to normal activities

Time to return to normal activities averaged 6.51 days in surgical group vs. 13.52 days in conservative group (Z = − 7.66, P < 0.001). There was obvious difference in two groups. (Table 3)

**Table 3:** *Time to return to normal activities* of Ligasure Hemorrhoidectomy and Conservative Treatment for TEH in pregnancy

	Ligasure hemorrhoidectomy (n = 35)	Conservative Management (n = 46)	*P* value
Time	6.51 ± 1.54	13.52 ± 2.49	< 0.001

### Post-treatment complications

The rate of post-treatment complications was 14.29% (5 cases) in surgical group and 26.09% (12 cases) in conservative group in Table 4. There was no obvious difference in two groups. In surgical group, two patients experienced urinary retention. They had to be catheterized overnight and were discharged the following day; two patients had uterine contraction and spontaneous relief without special treatment; one patient had mild fecal incontinence after 35 weeks of pregnancy and relieved after one month in postpartum. In conservative group, seven patients had to be hospitalized for persistent bleeding and threes of the cases underwent caesarean delivery due to pain or bleeding induced contractions.

**Table 4:** *Post-treatment complications* of Ligasure Hemorrhoidectomy and Conservative Treatment for TEH in pregnancy

	Surgical complications n (%)	Conservative complications n (%)	P value
Urine retention	2 (5.71%)	–	–
Bleeding	–	7 (15.22%)	–
Wound infection	–	–	–
Uterine contraction	2 (5.71%)	3 (6.52%)	0.629
Fecal incontinence	1 (2.86%)	–	–
Rectal stenosis	–	–	–
Total complications	5 (14.29%)	12 (26.09%)	0.196

### Pregnancy outcomes

Pregnancy outcomes were shown in Table 5. There were no significant differences between two groups for the rates of abortion, preterm birth, cesarean delivery, and the birth weight of fetus. There were no abortion patient in two groups. There were total 3 premature deliveries in surgical group who underwent cesarean delivery at 32–36 weeks of gestation: one for severe acute cholecystitis and two for twin pregnancy. 4/7 patients who received cesarean delivery were all related to obstetric disease. In conservative group, 3/5 premature deliveries underwent caesarean delivery due to pain or bleeding induced contractions. The birth weights of fetus were all within the normal range in two groups.

**Table 5:** *Pregnancy outcomes* of Ligasure Hemorrhoidectomy and Conservative Treatment for TEH in pregnancy

Variable	Ligasure hemorrhoidectomy (n = 35)	Conservative management (n = 46)	P value
Rates of abortion	0	0	–
Preterm birth	3 (8.57%)^a^	5 (10.87%)^a^	1.000
Cesarean delivery	7 (20%)^a^	10 (21.7%)^a^	0.849
Weight of fetus (g)	3100 (2564–3484)^b^	3150 (2522–3436)^b^	0.779

^a^n (%)

^b^Median (IQR)

### Recurrence

**Fig. 2:**
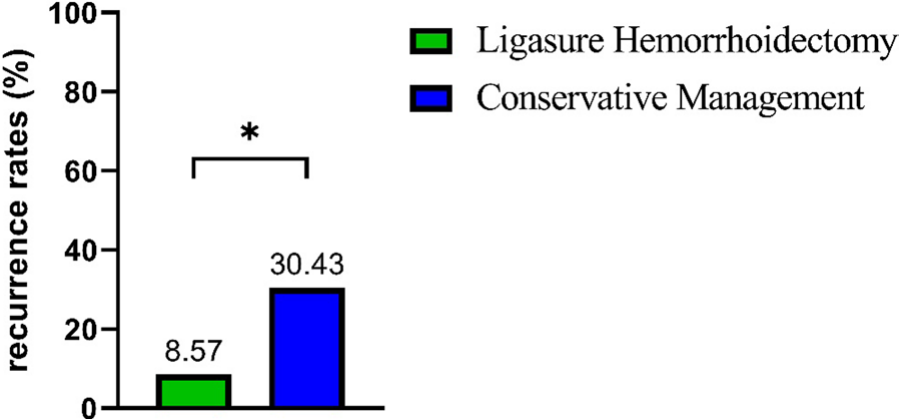
*Recurrence rates* of Ligasure Hemorrhoidectomy and Conservative Treatment for TEH in pregnancy. *Statistically significant

The overall recurrence rate at follow-up to 3 months postpartum was 20.99% (17 cases). The rate of recurrence in conservative group was 30.43% (14 cases), whereas only 8.57% (3 cases) in surgical group (χ^2^ = 5.73, P = 0.017, Fig. 2). 12/17 patients who had recurred appeared at birth delivery and 5/17 patients appeared within 7 days after delivery, 3 patients who had recurred in surgery group were in remission with conservative treatment, 5/14 patients who had recurred in conservative group were in remission with Ligasure hemorrhoidectomy.

### Patient satisfaction scores

Patient satisfaction scores were shown in Table 6. There was no statistical difference in patient satisfaction between two groups in 1week post-treatment. However, patient satisfaction scores were statistically significantly higher in surgical group than in conservative group at 2 weeks post-treatment and 3 months postpartum.

**Table 6:** *Patient satisfaction scores* of Ligasure Hemorrhoidectomy and Conservative Treatment for TEH in pregnancy

	Ligasure hemorrhoidectomy (n = 35)	Conservative management (n = 46)	*P*
1 week post-treatment	3 (2–4)	2 (1–3)	< 0.146
2 weeks post-treatment	3 (2–4)	1 (1–2)	< 0.001*
3 months after postpartum	3 (2–3)	2 (1.75–3)	0.019*

Results expressed as median (IQR); *Statistically significant

## Discussion

The results of our study indicate that Ligasure hemorrhoidectomy of TEH in pregnancy offers more rapid pain relief, shorter time to return to normal activities, lower incidence of recurrence, and better patient satisfaction. This type of surgery has low and mild postoperative complications, is not attended by any risk to the mother or her fetus. The Ligasure hemorrhoidectomy technique presented may be a feasible treatment for TEH in pregnancy.

The Ligasure vascular closure system is a new tissue coagulation device that has been gradually applied in hemorrhoidectomy. Ligasure hemorrhoidectomy removes the hemorrhoid tissue along the closed belt, and the vascular closure system achieves hemostatic effects by coagulation fibrin deformation. There is no need to ligate of the pedicle with silk suture. Less intraoperative bleeding, shorter operation time, less anal edema and swelling symptoms are obtained through this technique. Mild postoperative pain and no serious postoperative complications are conducive to the patient's rapid recovery to normal activities. Our results are encouraging in that Ligasure hemorrhoidectomy significantly resulted in mild postoperative pain and was associated with a fast return to normal activities, without serious postoperative complication, compare to conservative management. Similar to the study reported by Nienhuijs et al. [7]. So Ligasure hemorrhoidectomy offers an alternative to conventional hemorrhoidectomy.

In patients with TEH, treatment should be informed by shared decision-making, taking into account patient preferences, availability of procedures and fitness for further procedures [9]. Only careful selection of patients based on the severity of their symptoms and the choice of onset can bring about optimal treatment for any individual case. For such considerations, patients with TEH in pregnancy were offered Ligasure hemorrhoidectomy or conservative treatment. They were allowed to make their own choices among the options by themselves. We had found, the patients who had a prior history of TEH or with acute TEH symptoms sustaining less than 3 days preferred to choose Ligasure hemorrhoidectomy. The pregnancy patients with TEH who had initial onset with TEH or with TEH symptoms for a long duration time (more than 3 days tended to choose conservative treatment. In fact, they seeked conservative attention because of a fear of postoperative pain or they were afraid of surgery risk to their fetus.

There was significant difference in recurrence between two groups (8.57 vs. 30.43%, χ^2^ = 5.73, P = 0.017). Three patients who recurrence in surgical group were in remission with conservative treatment, 5 patients who recurrence in conservative group received Ligasure hemorrhoidectomy after postpartum. The result showed that conservative treatment might prolong the duration of symptoms and increased recurrence rates, and many patients would require subsequent radical operative treatment. Ligasure hemorrhoidectomy can confer protection against a further recurrence of TEH in pregnancy.

A fear of surgery or anesthesia risk to pregnancy and fetus is the surgeon’s biggest concern about surgery during pregnancy. Most of pregnancy patients with TEH seeked conservative treatment because of a fear of surgery or anesthesia risk to their fetus. There were no significant differences between the two groups for the rates of abortion, preterm birth, cesarean delivery, and the birth weight of fetus. The surgical group had mild post-treatment complications and safety pregnancy outcomes. It is also believed that the etiology of the underlying maternal illness that leads to the emergency surgery contributes to the poor pregnancy outcomes rather than the surgery itself [10].

The other controversy of surgery in pregnancy is the safety of anaesthesia. The ultimate goal of perioperative care of these patients is to provide safe anaesthesia to the mother while simultaneously minimising the (potential) risks of anaesthetics to the developing fetus [11].The major principles should include: (1) selecting anaesthetic drugs and techniques that have a good track record for safety; (2) employing regional anaesthesia whenever possible; (3) remembering that no anaesthetic agent or adjuvant drug has as yet been proven to be teratogenic in humans (this information should be transmitted to the patient prior to administering anaesthesia) [12]; In our study, patients in the surgical group received spinal anesthesia with 0.5% lidocaine in right lateral decubitus. Lidocaine is the most frequently used drug for Regional Anesthesia. Adverse effects and significant fetal consequence had not been found.

However, surgery is always very stressful and anxiety producing for the pregnant woman, even if the outcome is successful. It is important to keep in mind that physiologic changes during pregnancy alter the maternal response to stress. A thoughtful and comprehensive preoperative counsultation on the dangers to the fetus is an important component of therapy. Consultation and frequent communication with obstetrical colleagues and professional psychology are crucial. Fetal monitoring should be done throughout the perioperative period [13].

Constipation and a prior history of TEH may be the most common predisposing factors for TEH during pregnancy. The pathophysiology of constipation during the pregnancy is that progesterone causes a decreased small bowel and colonic motility and subsequent slow transit constipation, pressure on the rectosigmoid colon from the gravid uterus can cause an obstructive constellation of constipation symptoms [14]. As women with a history of TEH before the current pregnancy have more risk for developing TEH again, these women should be given extra attention and be instructed on how to prevent constipation. Hemorroid disease is a benign condition and its severity is not only related to the frequency of its symptoms but rather to how they are perceived by the patient. Indeed, similar symptoms may affect patients’ life style and quality of life in very different ways with a significant variation from patient to patient. The patient satisfaction should be considered when assessing the effects of the therapeutic approach [15]. This questionnaire is a more comprehensive assessment of patient satisfaction. It is the first time to evaluate therapy efficacy and safety including patient satisfaction for TEH in pregnancy.

Our study had some limitations. First, this was a retrospective study with a limited number of patients, the data was obtained from only one center. Thus, selection bias was inevitable. Another limitation was the short length of follow-up. The result could not accurately evaluate recurrence rate. Finally, two anorectal surgeons took part in the management of these patients, and their individual decisions and surgical techniques might affect the results. Therefore, further well-designed randomized studies are required.

## Conclusions

Compared with conservative treatment, Ligasure hemorrhoidectomy of TEH in pregnancy results in more rapid pain relief, shorter time to return to normal activities, lower incidence of recurrence, and better patient satisfaction. This type of surgery has low and mild postoperative complications, is not attended by any risk to the mother or her fetus.

## Data Availability

The datasets of the current study are available from the corresponding author upon reasonable request.
